# Psychiatric associations’ response to climate change-driven wildfires and disaster preparedness

**DOI:** 10.1192/j.eurpsy.2026.10383

**Published:** 2026-07-31

**Authors:** H. Gazey

**Affiliations:** Psychiatry, Bakirkoy Prof. Dr. Mazhar Osman Research and Training Hospital For Psychiatry, Neurology and Neurosurgery, Istanbul, Türkiye

## Abstract

**Abstract:**

Climate change has contributed to an increase in the frequency and intensity of extreme weather events such as droughts, floods, and wildfires. Literature reported that that climate change contributed to approximately 93% of the 152 extreme heat events studied, 56% of the 126 precipitation or flood events, and 68% of the 81 drought events. Similarly, climate change has been shown to contribute to an increase in the area affected by wildfires in seasonally dry forests due to rising seasonal temperatures, prolonged dry summer periods, low winter precipitation, and early snowmelt.

Climate change is a global crisis that affects mental health both directly and indirectly. Extreme heat, air pollution and natural disasters create biological and psychological stress, while secondary effects such as displacement, economic loss and uncertainty are associated with increased depression, anxiety, post-traumatic stress disorder and substance use. Individuals with serious mental illness are at higher risk due to both their physiological vulnerability and social disadvantages. In this respect, the climate crisis is a structural public health issue that requires psychiatric associations to redefine their scientific, ethical, and social responsibilities.

Among these institutional responsibilities are the development of evidence-based guidelines, training modules, and disaster preparedness protocols addressing the effects of climate change on mental health, and the standardisation of clinical recommendations for protecting individuals with serious mental disorders from risks such as heat waves, air pollution, and displacement; taking steps to establish emergency plans that will ensure continuity of care in health institutions, presenting frameworks for telepsychiatry and community-based care models, and carrying out public policy activities that focus on mental health.

**Disclosure of Interest:**

None Declared

